# Gas-Phase Synthesis for Label-Free Biosensors: Zinc-Oxide Nanowires Functionalized with Gold Nanoparticles

**DOI:** 10.1038/s41598-019-53960-2

**Published:** 2019-11-22

**Authors:** E. Danielson, V. Dhamodharan, A. Porkovich, P. Kumar, N. Jian, Z. Ziadi, P. Grammatikopoulos, V. A. Sontakke, Y. Yokobayashi, M. Sowwan

**Affiliations:** 1 Nanoparticles by Design Unit, Okinawa Institute of Science and Technology (OIST) Graduate University, 1919-1 Tancha, Onna-Son, Okinawa 904-0495 Japan; 2 Nucleic Acid Chemistry and Engineering Unit, Okinawa Institute of Science and Technology (OIST) Graduate University, 1919-1 Tancha, Onna-Son, Okinawa 904-0495 Japan; 3 Institute of Nanosurface Science and Engineering (INSE), Shenzhen University, Shenzhen, 518060 China

**Keywords:** Sensors and biosensors, Nanoparticles, Nanowires

## Abstract

Metal oxide semiconductor nanowires have important applications in label-free biosensing due to their ease of fabrication and ultralow detection limits. Typically, chemical functionalization of the oxide surface is necessary for specific biological analyte detection. We instead demonstrate the use of gas-phase synthesis of gold nanoparticles (Au NPs) to decorate zinc oxide nanowire (ZnO NW) devices for biosensing applications. Uniform ZnO NW devices were fabricated using a vapor-solid-liquid method in a chemical vapor deposition (CVD) furnace. Magnetron-sputtering of a Au target combined with a quadrupole mass filter for cluster size selection was used to deposit Au NPs on the ZnO NWs. Without additional functionalization, we electrically detect DNA binding on the nanowire at sub-nanomolar concentrations and visualize individual DNA strands using atomic force microscopy (AFM). By attaching a DNA aptamer for streptavidin to the biosensor, we detect both streptavidin and the complementary DNA strand at sub-nanomolar concentrations. Au NP decoration also enables sub-nanomolar DNA detection in passivated ZnO NWs that are resilient to dissolution in aqueous solutions. This novel method of biosensor functionalization can be applied to many semiconductor materials for highly sensitive and label-free detection of a wide range of biomolecules.

## Introduction

DNA detection has many applications in clinical diagnostics and biomedical research, prompting development in a large variety of methods to detect small quantities of specific DNA sequences^1,2^. Nucleic acid sequences can also be developed into artificial DNA aptamers, designed to bind to biomolecules of interest for early disease detection^3^. Optical methods are typically used for DNA sensing applications, but these approaches require labeling the relevant DNA sequence with fluorescent molecules, as well as sophisticated photodetectors^4,5^. Therefore, there is a strong need to develop label-free methods of DNA detection that are simple, low-cost, and highly sensitive.

Nanomaterials, especially one-dimensional semiconductor nanowires, have demonstrated their capability as label-free sensors for a variety of biomolecules, including DNA^6–11^. When a charged molecule binds to the surface of semiconductor nanowire, the charge density of the nanowire is altered, causing a change in its conductance^6^. Due to the nanowire’s high surface to volume ratio, the binding of a small number of biomolecules can produce a large electrical signal easily measurable with macroscopic instruments^10,12,13^.

The selectivity of a sensor describes its ability to respond to the presence of a specific analyte when other interfering substances are present. Normally, the selectivity of nanowire biosensors is defined by immobilizing receptor molecules on the nanowire surface to uniquely bind to the targeted analyte, such as a complementary DNA strand. Many semiconducting nanowires (e.g. Si, ZnO, In_2_O_3_) have a surface oxide layer which can be treated to generate functional amine, thiol, or aldehyde groups for covalent attachment of receptor molecules^7,14^. However, this surface treatment procedure can alter the charges on the nanowire surface and subsequently change its conductance. In addition, the presence of this layer increases the effective insulating thickness between the charged analyte and the nanowire, degrading the sensor’s detection limit.

In this study, we instead functionalize nanowires using gold nanoparticles (Au NPs) deposited via a gas-phase synthesis method. Nanoparticles have been employed as selective transducers for a wide variety of biosensor types^15–17^. Gold especially is biocompatible and has high chemical affinity with thiol groups, enabling easy binding of thiol-terminated DNA and improving binding selectivity^12^. We employ a magnetron-sputter gas aggregation method to synthesize and deposit Au nanoparticles on the nanowire sensors, which has not been used previously for biosensor functionalization. This method allows for a high degree of deposition parameter control in order to ensure consistency in nanoparticle size, microstructure, and area coverage^18–21^. Magnetron sputtering also avoids the complications in solvent-based nanoparticle synthesis methods that may result in residual capping ligands or reactants that could interfere with the presentation of a bare Au surface for thiol binding^22,23^.

**Figure 1:**
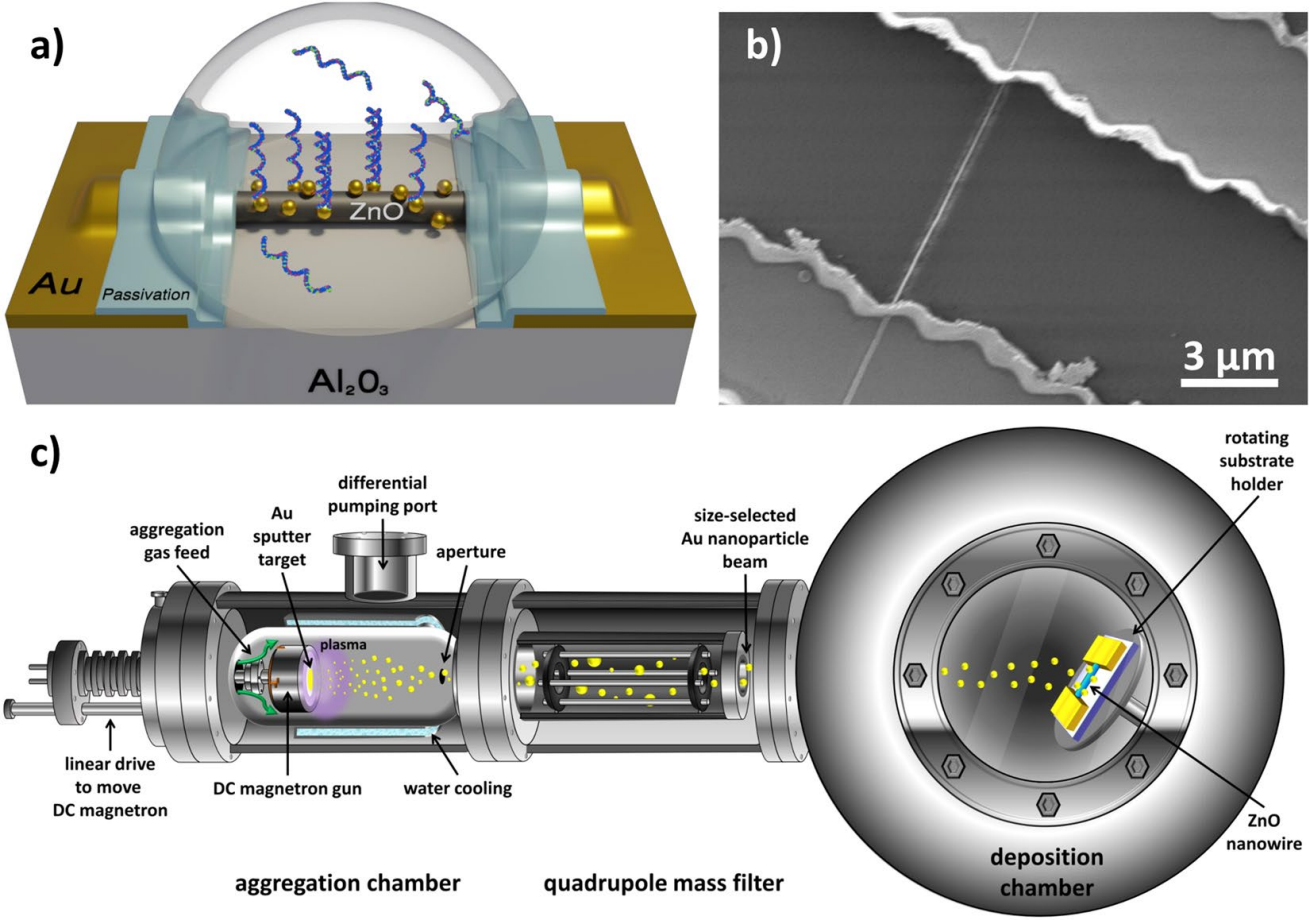
ZnO NW Device & Gas-phase synthesis of Au NPs (**a**) Schematic diagram of a passivated ZnO NW biosensor with Au NP decoration for DNA sensing in solution. *Created by Pavel Puchenkov using Blender 2*.*8;* www.blender.org (**b**) SEM image of a ZnO NW between Au electrodes before Al_2_O_3_ passivation layer deposition. (**c**) Schematic diagram of the magnetron-sputtering system for gas-phase synthesis and deposition of size selected Au NPs. *Created using Microsoft Office Professional Plus 2010; products*.*office*.*com/home*.

Zinc oxide is a widely used material in nanowire biosensor research due to its high isoelectric point, non-toxicity, and biocompatibility^24,25^. We grow ZnO NWs using a vapor-liquid-solid growth process, organize them into amperometric biosensors, then decorate them with Au NPs formed using our gas-phase synthesis technique (Fig. 1). The nanowire sensor is then tagged with a single-stranded DNA aptamer that can selectively bind to either its complementary strand (cDNA) or streptavidin, a high-affinity protein that is commonly used in biosensing research^26–30^. As ZnO is an n-type semiconductor, both streptavidin (negatively charged in neutral pH solutions) and DNA (negatively charged due to the phosphate backbone) binding will deplete charge carriers in the nanowire and decrease conductance.

From this conductivity change, we demonstrate sub-nanomolar detection of both analytes in Au NP decorated ZnO NW devices. Undecorated NW devices show no change in conductivity over short timescales, but degrade after extended aqueous solution exposure, artificially mimicking the conductivity response of a negatively charged analyte. By passivating the NW and decorating with Au NPs, we fabricate robust ZnO NW devices that retain their sub-nanomolar detection sensitivity to DNA. This functionalization method limits potential false responses due to ZnO NW degradation, which has likely been underestimated in the literature.

## Results and Discussion

### Transmission electron microscopy (TEM) studies

**Figure 2:**
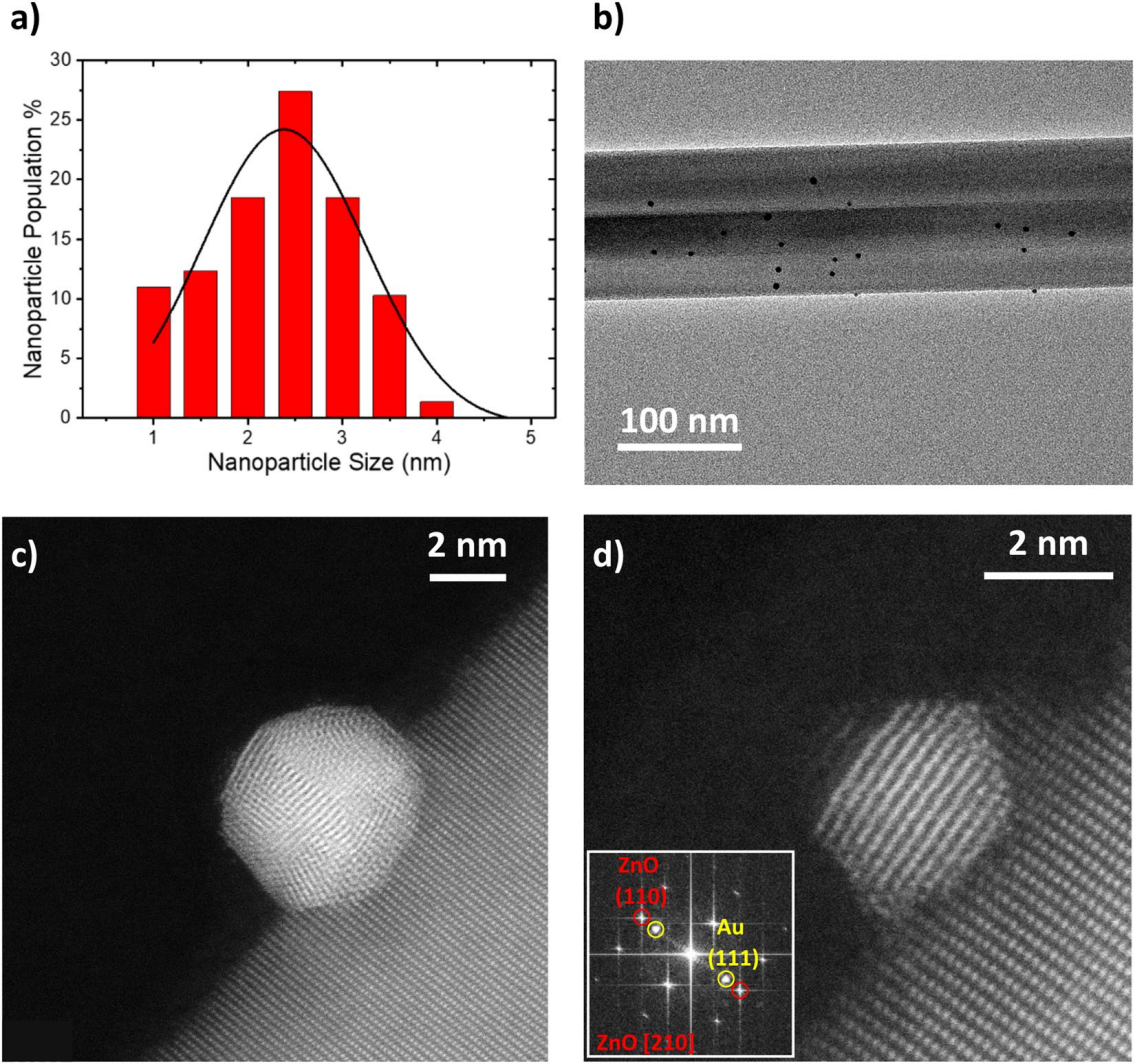
TEM Characterization of Au NPs. (**a**) Particle size histogram (red) of sputtered Au NPs on a Si_3_N_4_ TEM grid (deposited simultaneously with ZnO NW biosensors). Particle diameter was measured and fitted with a Gaussian curve (black). (**b**) Low magnification TEM image of ZnO NW decorated with Au NPs. (**c**) HAADF-STEM image of a polycrystalline Au NP on the surface of a ZnO NW. (**d**) HAADF-STEM image of a single crystal Au NP on the surface of a ZnO NW. The inset FFT of the image shows spots corresponding to crystal planes from the ZnO NW (red) and Au NP (yellow). For the single crystal Au NP in (**d**) the Au (111) planes appears parallel to the (110) planes of ZnO.

The Au NPs size distribution, shown in Fig. 2a, was obtained from TEM observation. The distribution shows that the average nanoparticle diameter size is 2.5 nm, in agreement with the QMF selection during deposition. The gas phase synthesis method displayed good control of the Au NPs size (Fig. 2b), while allowing the flexibility of changing coverage over the device by modifying the deposition time. The size distribution was chosen to maximize the number of binding sites while minimizing the chance for DNA strand tangling after binding. Thus, optimizing the performance of the biosensor. Size control of the nanoparticles is also necessary so that DNA binds to the Au NP within the Debye length of the solution. Outside of this distance, the influence of the charged biomolecules on the semiconductor nanowire will be electrically screened by counterions in the solution^31^. In this work, DNA or streptavidin was prepared in 0.01x phosphate buffered solution (PBS, ph 7.2–7.4) with a Debye length of 7.2 nm. Size selection in the gas phase and soft landing of the Au NPs ensures that biological molecules will be bound within this distance to the ZnO NW.

Figures 2c,d show HAADF-STEM images of stable gas-phase synthesized Au NPs anchored on highly crystalline ZnO NWs grown by CVD. Interplanar distances were measured from both images to be 0.25 nm and 0.15 nm, which were identified as the (002) and (110) crystal planes, respectively, of ZnO. Thus, it can be shown that the ZnO is orientated onto the [210] zone axis. The majority of Au NPs deposited using gas-phase synthesis are polycrystalline and exhibit limited crystal plane association with the underlying hexagonal wurtzite structure of the ZnO NW (Fig. 2c). Single crystal Au NPs, in contrast, show parallel plane alignment between the Au (111) plane and the ZnO (110) plane, as indicated in the Fast Fourier Transformation (FFT) inset in Fig. 2d. It is also clear from the HAADF-STEM images that the nanoparticle synthesis method does not damage the ZnO NW, which retains its single crystallinity necessary for high conductivity. The crystalline surface also provides a good contact interface for the deposited Au NPs. Furthermore, by gas-phase Au NP synthesis, we ensure clean binding for the thiol DNA aptamer without additional surface modification of the biosensor.

### Atomic force microscopy measurements

**Figure 3:**
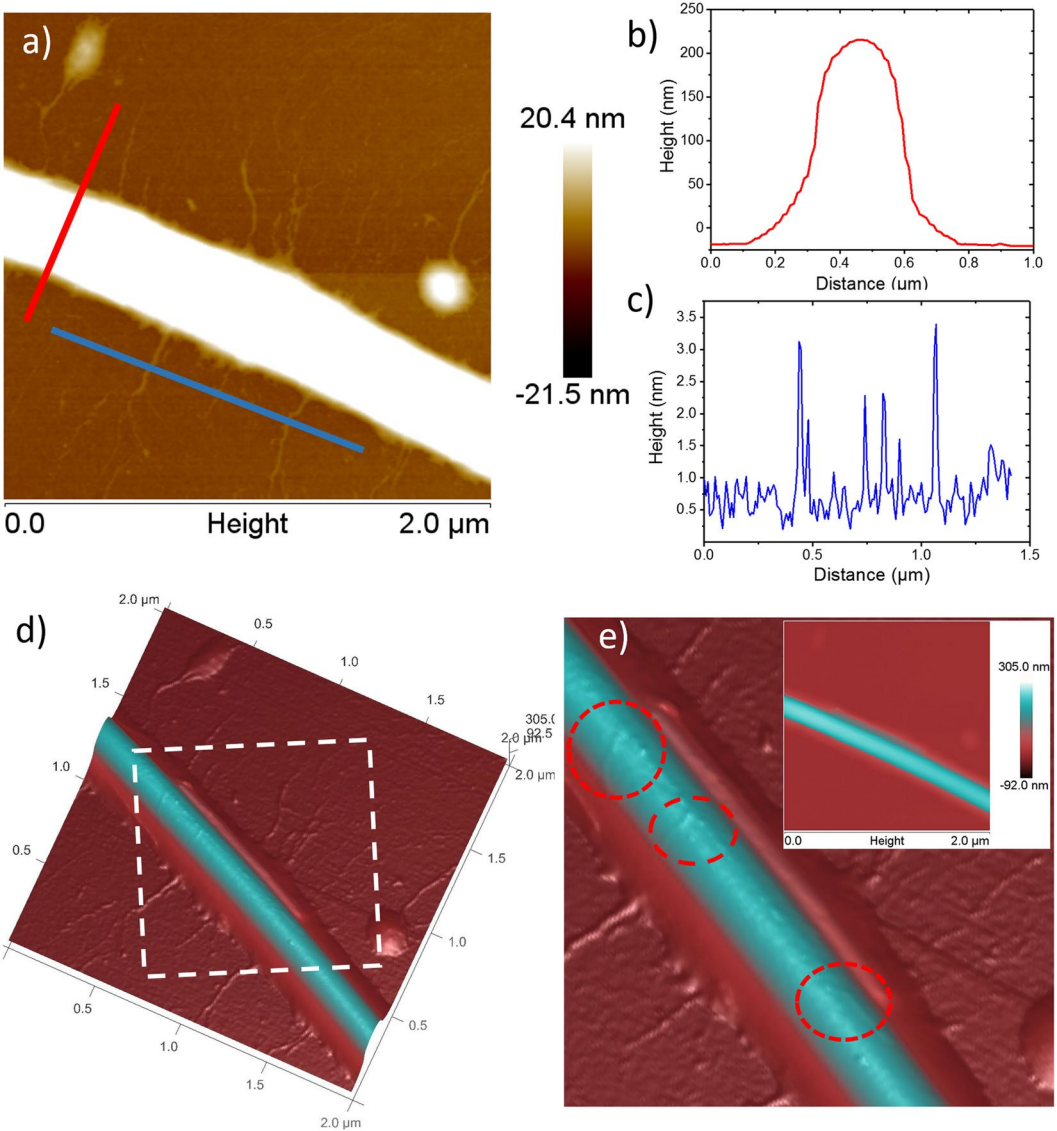
Visualization of DNA attachment through AFM. (**a**) AFM image of a ZnO NW decorated with Au NPs showing attachment of duplex DNA at specific sites. Height profiles of the nanowire (**b**) and duplex DNA strands (**c**) on the SiO_2_ substrate. (**d**) AFM 3D image of (**a**); here the color is adjusted to see the surface of the ZnO NW. The area enclosed by the white dotted line is enlarged in (**e**) while the inset in (**e**) is the 2D AFM image with adjusted color scale. Au NPs attached to the ZnO NW surface are highlighted using red dotted lines in (**e**).

Decorated ZnO NWs on an Al_2_O_3_ substrate were mechanically transferred to a Si substrate via scratching, then 40 µL of a 10 µM duplex thiolated DNA solution was drop cast onto the substrate and left under ambient conditions for 1 hour. The covalent bond between gold and sulfur strongly attaches the DNA strand to Au NPs along the nanowire. These thiol bonds are commonly used in biosensing, drug delivery, and molecular biology studies for the functionalization of gold surfaces for sensing and attaching individual biomolecules to gold^32^. The substrate was washed with 0.5x TBE buffer solution to remove unattached DNA strands and dried under a N_2_ gas purge. In Fig. 3, duplex DNA is attached to the nanowire at isolated and dispersed locations, like the distribution of Au NPs seen in Fig. 2b. The duplex DNA used in this experiment was 2000 bp, or ~680 nm, in length so that it would be easily visible. The height profiles of the nanowire and individual DNA strands are also shown Fig. 3b,c. Figure 3d shows the AFM 3D image of the same ZnO NW with adjusted image color scale in order to see the surface of the ZnO NW. The area indicated by a white dotted line is shown again in Fig. 3e, with corresponding 2D image of Fig. 3d as an inset. From the enlarged image in Fig. 3e we can clearly see isolated Au NPs attached to the DNA.

The AFM profile of the DNA strands, approximately 2 nm, are consistent with the binding of one duplex DNA strand per Au NP site. Without using previous ZnO surface modification methods or treatments we were able to attach individual DNA strands to nanowires solely using magnetron-sputtering. Synthesizing the nanoparticles using a gas-phase method allows us to control the number of potential binding sites on each nanowire. Through size selection of the Au NPs, we also control the number of DNA strands bound on each Au NP site, limiting strand tangling and self-binding events in solution that limit the sensitivity of the NW biosensor.

### Electrical sensing measurements

**Figure 4:**
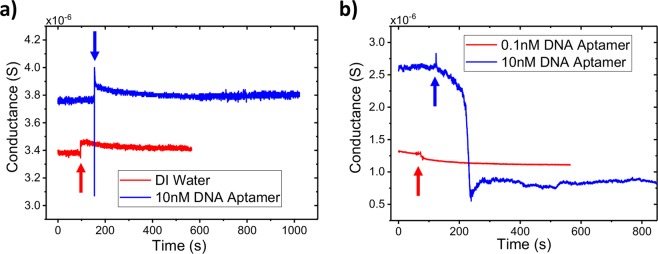
Biosensor response to thiolated DNA aptamer Conductance vs. time plots of bare (**a**) and Au NP decorated (**b**) single ZnO NW devices upon introduction (arrows) of 10 µL of the indicated solution. The negatively charged thiolated DNA aptamer binds to the Au NPs on the NW surface, inducing positive charges and decreasing the ZnO NW conductivity; in contrast, DNA is unable to bind to the unmodified NW surface and solution introduction has no effect on conductivity.

We compare the change in conductance of bare and Au NP decorated ZnO NW devices upon introduction of a 10 nM solution of the DNA aptamer in dark ambient conditions (Fig. 4). The undecorated, bare ZnO NW device shows a small increase in conductance, but gradually returns to the initial value. This behavior is similar to the response of an undecorated NW device to DI water. The thiolated DNA aptamer is unable to bind to the unmodified surface of the ZnO NW and affect the NW conductivity. In contrast, the decorated NW device shows an immediate decrease in conductance, as negatively charged DNA depletes charge carriers in the n-type ZnO. This decrease in conductance occurs on short time scales due to the strong binding kinetics of the gold-thiol interaction, in contrast to other methods of ZnO surface modification, which require longer periods of incubation^33^. As the binding sites on the ZnO NW become saturated with DNA, the conductance stabilizes at a reduced value proportional to the DNA solution concentration. Accordingly, a low concentration 100 pM DNA solution results in a much smaller conductance change (Fig. 4b).

**Figure 5:**
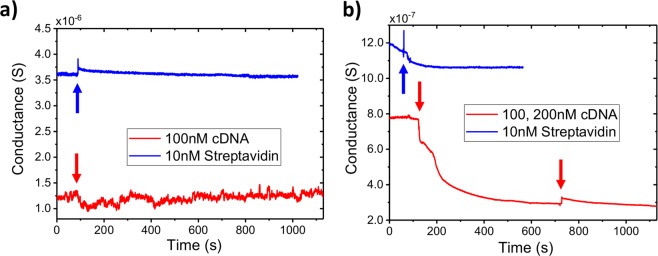
Biosensor response to different analytes. Conductance vs. time plots of bare (**a**) and Au NP decorated (**b**) single ZnO NW devices upon introduction (arrows) of 10 µL of the indicated solution. Au NP decorated biosensors respond to multiple analytes after functionalization with the same DNA aptamer. Increased analyte concentration results in a proportionally larger change in Au NP decorated NW conductivity, until saturation of all available Au NP sites.

We characterized the DNA aptamer functionalized sensor by measuring the conductance response to solutions of the complementary (cDNA) strand and streptavidin, a common model protein system for biosensors^30^. By functionalizing our sensors with a 60 nt DNA aptamer for streptavidin, we show the utility of Au NP decorated devices for detecting different types of biomolecules in a solution simultaneously. After DNA functionalization with a 10 nM solution, the devices were washed with 1x PBS and DI water to remove weakly bonded DNA aptamer strands. The decorated and undecorated ZnO NW devices were exposed to solutions of 10 nM streptavidin and 100 nM of the cDNA (Fig. 5). As before, there is no change in the undecorated NW device conductance, as the streptavidin and cDNA do not react with the bare ZnO surface (Fig. 5a). Using the Au NP-decorated device, both streptavidin and the cDNA bind to the DNA aptamer attached to the decorated ZnO surface. As both are negatively charged at neutral pH, the binding of these analytes results in a depletion of charge carriers in the n-type ZnO and therefore a decrease in the NW conductance (Fig. 5b). The higher concentration of the cDNA solution, as well as the larger negative charge of DNA compared with streptavidin at neutral pH, results in a larger decrease in conductance, but application of a 200 nM cDNA solution did not produce an additional decrease. As the NW sensor was exposed to a solution with 10 nM of the DNA aptamer, the high concentration of the cDNA solution rapidly saturates all available Au NPs with attached DNA strands.

In order to determine the concentration dependence and minimum sensitivity of the Au NP-decorated ZnO biosensor, we systematically measured the output I-V currents under different target cDNA concentrations after exposure to a 10 µM solution of the DNA aptamer for 10 min, using methods adopted from the literature^34,35^. Current-voltage measurements were taken after 10 min of application with 10 µL of solutions with 100 pM, 1 nM, and 10 nM of cDNA. The NW devices were washed after the initial functionalization step but not between analyte solution applications. This was done to limit the solution exposure time of the ZnO NWs, which are known to be chemically unstable in aqueous solutions^14,36–41^.

**Figure 6:**
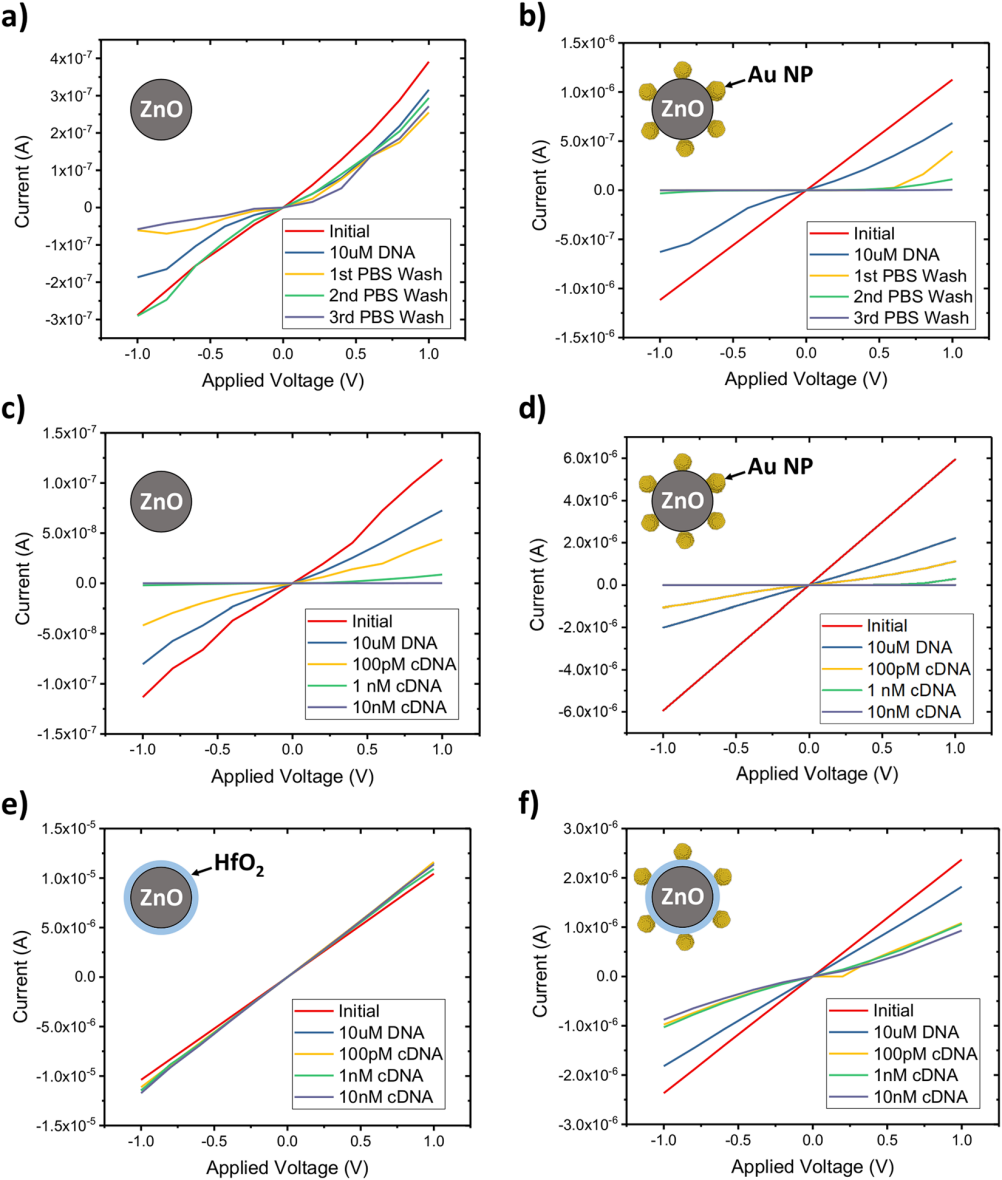
Similarity of degradation and binding response I-V curves of nonfunctionalized (**a**,**c**,**e**) and Au NP (**b**,**d**,**f**) decorated ZnO NW sensors after being exposed to 1x PBS solutions or different DNA concentrations. ZnO degradation from aqueous solution exposure produces sensor responses difficult to differentiate from negatively charged analyte binding (**a**–**d**). HfO_2_ passivation (10 nm via ALD) limits NW conductivity change with solution exposure (**e**) and Au NP decoration preserves sensitivity to DNA binding and hybridization (**f**).

Previous results have indicated that ZnO NWs are stable in biological solutions for several hours^41^, however we found that exposure to these cDNA solutions severely impacted the nanowire device performance on the timescale of several minutes. Upon exposure to cDNA solutions, as well as in control experiments using 1x PBS, unprotected ZnO NWs show physical degradation and decreased conductance (Fig. 6a–d). Both decorated and undecorated nanowires with thicknesses up to 200 nm were completely dissolved after less than 1 hour of aqueous solution exposure. Repeated exposure of control solutions caused the nanowire conductance to decrease in a manner superficially similar to the effect of high concentration cDNA or streptavidin binding^35^. We find that partial chemical dissolution of ZnO in aqueous solutions on short timescales can mimic the electrical response of a negatively charged analyte binding to the nanowire. This limits the applicability of ZnO even in the case of biocompatible one-time-use sensors. These results emphasize the need for control experiments in metal oxide biosensor research and the development of a method to prevent ZnO degradation^40^. Larger ZnO ‘nano’-wires (>500 nm diameter) may be also be useful due to their increased durability, however their size may limit sensitivity to some biomolecules^6^.

To mitigate this issue, we deposited a 10 nm passivation layer of HfO_2_ over the device, without leaving a solution ‘window’ for the nanowire. This allowed ZnO NW devices to be stable in PBS solution for several hours and undergo several washing procedures without decreasing conductivity. Au NPs were deposited in the same manner on HfO_2_-sheathed ZnO NW devices. HfO_2_ was chosen for its inertness as well as its high-κ dielectric properties so that electrically charged molecules could still deplete charges and change the conductivity in the sheathed nanowire^42^. We found that 5 nm thick layers did not prevent dissolution and thicker HfO_2_ films yielded completely unresponsive devices. However, at 10 nm passivation layer thickness, sensitive device fabrication yield did decrease significantly. Representative 10 nm HfO_2_ passivated device performance with respect to analyte concentration are presented in Fig. 6e,f. Undecorated ZnO NW devices show no change in their Ohmic I-V behavior upon exposure to thiol-terminated DNA aptamer, or the cDNA. In contrast, the Au NP-decorated nanowire is initially Ohmic in its I-V behavior, but attachment of the negatively charged DNA aptamer results in a depletion of the electron carriers in the nanowires and its behavior becomes more Schottky-like^43,44^. As the cDNA is introduced, these hybridize with the attached strands of the DNA aptamer and deplete more charge carriers, increasing the Schottky barriers at the source and drain electrodes. Although the biosensor is highly sensitive to the initial introduction of the complementary strand (~1 µA current drop at 100 pM), raising the cDNA concentration by orders of magnitude does not create a proportional response; due to the number of Au NP binding sites being much smaller and more easily saturated than in a SAM-functionalized nanowire. This series of tests was repeated using streptavidin as an analyte. With functionalization of the Au NP-decorated ZnO NW with 10 uM of the DNA aptamer, the biosensor is capable of detecting both streptavidin and the complementary DNA strand at 100pM. This sensitivity is comparable to or an improvement on previously published ZnO biosensors that utilize a self-assembled monolayer (SAM) functionalization method^27,28,30,35^. The use of well-known Au NP biochemistry allows for a wide range of different potential applications of this functionalization method with other DNA aptamers or biomolecules.

**Figure 7:**
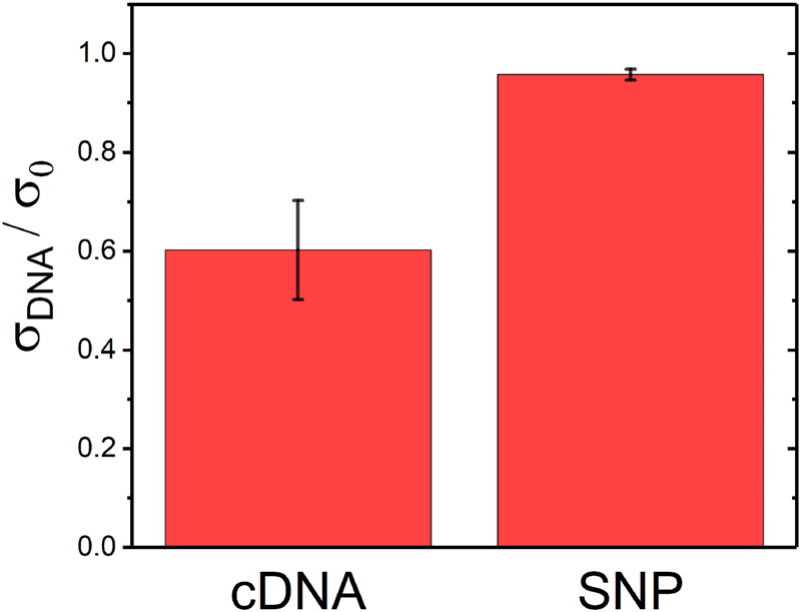
Biosensor Selectivity. Conductivity change in Au NP-decorated ZnO NW biosensors upon introduction of a 10 nM solution of a fully complementary DNA strand (cDNA) or a strand containing a single nucleotide polymorphism (SNP). Fully complementary strands induce a larger decrease in the ZnO NW conductivity. Error bars show the standard deviations taken from at least five samples.

These passivated ZnO NW devices also show good analyte selectivity and minimally respond to DNA that is not complementary to the aptamer. The selectivity was tested by measuring the ZnO NW conductivity after introduction of a solution containing DNA with a single nucleotide polymorphism (SNP) relative to the aptamer. As shown in Fig. 7, there is little conductivity change due to the SNP DNA, as it is weakly bound to the DNA aptamer; unzipping and washing away before measurement. The hybridization is specific to the complementary strand and the biosensor can distinguish the cDNA from SNPs due to the proportionally larger reduction in conductivity.

In summary, a ZnO NW device was fabricated for use as a biosensor for DNA and streptavidin, using a DNA aptamer. The deposition of Au NPs onto these devices using a magnetron-sputtering method allows for these devices to be used for biosensing applications without the need for chemical functionalization of the NW surface. Au NP-decorated NW devices can detect both streptavidin and DNA at sub-nanomolar concentrations, with high magnitude current responses and conductivity changes. This functionalization method allows the use of well-known Au NP chemistry to simultaneously detect a wide range of different biomolecules using label-free ZnO biosensors. By controlling the nanoparticle deposition parameters, a limited number of binding sites can be created for highly sensitive devices. Size control of the nanoparticles also limits false positive self-binding events. However, nanowire degradation on short timescales in aqueous solutions can lead to signals similar to negatively charged analyte binding, as ZnO is an n-type semiconductor. This effect has been underestimated in the literature and highlights the necessity of ZnO surface treatment for stable devices. Alternatively, more stable biosensor nanostructures could be employed to take advantage of this efficient functionalization technique.

## Methods

### Zinc oxide nanowire sensor fabrication

Zinc oxide nanowires (ZnO NWs) used in this study were prepared through a vapor-solid-liquid growth process. ZnO nanopowder and graphite powder were mixed in a 1:1 ratio and transferred to a carbon boat placed inside a chemical vapor deposition (CVD) tube furnace. Al_2_O_3_ (sapphire) substrates were also placed inside the tube, approximately 15 cm away from the carbon boat in the direction of gas flow. Previously, 2 nm of gold was thermally deposited onto the Al_2_O_3_ substrates in a vacuum chamber, and then patterned using photolithography to act as nucleation sites. The tube furnace was heated to 940 °C under a flow of 60 sccm Ar and 20 sccm O_2_ for 2 hours. The Au layer melts and condenses into droplets which serve as preferential catalyst sites for the vapor reactant^45^. Typical ZnO NW thickness was 50 to 200 nm and the nanowires grew to an average length of 20 µm from the nucleation sites along the [1 $\bar{1}$ 0] direction of the Al_2_O_3_ substrates. Nanowire structure and positions were examined using an FEI Quanta 250 scanning electron microscope (SEM). Single nanowire devices were fabricated by depositing 5/50 nm of Ti/Au via e-beam evaporation over the ZnO NW to act as source and drain electrodes (Fig. 1b). For devices used in real-time liquid sensing experiments, a 20 nm layer of Al_2_O_3_ was deposited as a passivation layer over the electrodes using an atomic layer deposition (ALD) system. A 5 µm long segment of the nanowire was protected with photoresist during the ALD step and exposed during liftoff to create a sensing window.

### Gold nanoparticle decoration

ZnO NW devices were then decorated with Au NPs using a DC magnetron-sputtering inert-gas aggregation system (Fig. 1c)^18–21^. A high purity (99%) gold target is bombarded with an Ar plasma, freeing Au atoms for nanocluster formation. A controlled pressure difference between the particle aggregation zone and deposition chamber allows us to control the nanoparticle size and crystallinity. Before deposition the base pressure was ~1.5 × 10^–7^ mbar, whereas during deposition the pressures were ~1.4 × 10^−3^ and ~4.5 × 10^−1^ mbar in the deposition chamber and in the aggregation zone, respectively. Depositions were performed using an Ar flow rate of 100 sccm, an operating magnetron power of ~6 W and an aggregation length of 125 mm. The particles also pass through a quadrupole mass filter (QMF) before deposition for size control. We selected Au NPs with an average diameter of 2.5 nm to minimize instances of multiple DNA strands binding to a single nanoparticle. Under these deposition conditions for 1 hour, the nanoparticle density is approximately 5 × 10^10^ cm^−2^, therefore each ZnO NW device will present >100 binding sites to the biological solution. The rotation speed of the substrate holder was set at 2 rpm to ensure uniform device coverage.

### Characterization

Atomic force microscopy (AFM) was used to verify the attachment of double stranded (duplex) DNA to the ZnO NWs. The AFM topography measurements were performed on a conventional Multimode 8 scanning probe microscope (Bruker, USA) in Peak Force tapping mode. The high-resolution AFM probe (ScanAsyst-Air) from Bruker having nominal tip radius ~2 nm, resonant frequency 70 kHz and low spring constant 0.4 N/m were used for all AFM measurements. The high resolution (512 × 512 pixels) AFM images were captured at a scan rate of 0.5 Hz and further processed by using the Nanoscope Analysis software (Ver 9). High Angle Annular Dark Field Scanning Transmission electron microscopy (HAADF-STEM) was used to study Au NP and ZnO NW surface interaction. For STEM imaging, Au NP decorated ZnO NWs were mechanically transferred to holey carbon coated gold mesh TEM grids and imaged using a JEOL JEM-ARM 200 F STEM equipped with a probe spherical aberration corrector (operation voltage 200 kV). The collection angle of the HAADF detector is from 68 to 280 mrad.

### Biological solution preparation

The dimeric form of the streptavidin-binding aptamer was derived from the monomeric form (StrepApt5) reported by Ruigrok VJ *et al*.^46^.

Aptamer: 5′-thiol-modified/AAAGGGAACGCACCGATCGCAGGTTTCCCATAAACACGACGCACCGATCGCAGGTTCGTG-3′ (60 nt)

Complementary strand (cDNA): 5′-TTTATGGGAAACCTGCGATCGGTGCGTTCCCTTT-3′ (34 nt)

Non-complementary strand (SNP): 5′-TTTATGGGAAAGCTGCGATCGGTGCGTTCCCTTT-3′ (34 nt)

The disulfide in a thiol-modified aptamer solution was reduced to monothiol using tris (2-carboxyethyl) phosphine (TCEP 20 mM, 2 hrs at RT). A solution containing 1x TBE buffer (89 mM Tris, 89 mM boric acid, 2 mM EDTA, pH ~8.0), 1 µM TCEP, and 50 mM NaCl was used for cleaving the s-s bond of the 10 nM aptamer. This solution was kept at −20 °C. The following oligonucleotides were used for preparing 2000 bp DNA with 5′-thiol modification.xx

PCR primer 1: 5′-thiol-modifed/GTCTCGCGCGTTTCGGTGAT-3′ (20 nt)

PCR primer 2: GAACCGGAGCTGAATGAAGCC-3′ (21 nt)

The 2000 bp DNA with 5′-thiol modification was prepared by PCR amplification of the pUC19 plasmid. The plasmid (10 ng) with primers 1 and 2 (0.5 µM each) were PCR amplified using 2 × Phusion Master Mix (New England Biolabs) in 25 µl reaction volume. After initial denaturation at 98 °C for 30 sec, 25 cycles of 5 sec at 98 °C followed by 45 sec at 72 °C and 30 sec at 70 °C were used. The resulting PCR product was column purified using a DNA Clean and Concentrator Kit (Zymo Research). A solution containing 3 nM of thiol-modified duplex DNA was subjected to TCEP treatment as mentioned previously. Streptavidin (10 nM) solution was prepared in 1x phosphate-buffered saline (PBS) buffer (137 mM NaCl, 2.7 mM KCl, 10 mM Na_2_HPO_4_, 1.8 mM KH_2_PO_4,_ pH~7.4).

### Electrical measurements

The devices were measured in a probe station under ambient conditions using a Keithley 6221 Current Source and 2182 A Nanovoltmeter to perform low voltage differential conductance measurements. An alternating current with amplitude ± 10 nA is synchronized with the nanovoltmeter to measure the voltage at each current step, then calculating the delta voltage between consecutive steps. Each delta voltage is averaged with the previous one to calculate the differential voltage, dV, which is then used to derive the differential conductance ΔG from ${dI}/{dV}$. This method significantly reduces electrical noise and compensates for changes in the conductance over time due to thermoelectric effects. Current-voltage (I-V) measurements were also performed in the probe station under ambient conditions using a Keithley 2636 A with applied voltages from ±1 V. Solutions of DNA or streptavidin were diluted to nanomolar concentrations using 0.01x phosphate buffered solution (PBS, pH 7.2–7.4) in order to maintain a Debye length of 7.2 nm so that the influence of the charged biomolecules would not be electrically screened by the solution^31^. During conductance measurements, 10 µL of the analyte solution was dripped onto the surface of the ZnO NW device under ambient conditions. Concentration dependent experiments were conducted by keeping the droplet on the NW device for 10 min, then rinsing with 1x PBS and deionized (DI) water before I-V measurements.
